# The EMazing Race: A Novel Gamified Board and Clinical Practice Review for Emergency Medicine Residents

**DOI:** 10.21980/J8.52075

**Published:** 2025-10-31

**Authors:** Brendan Freeman, Kevin Vinokur, Michael Zimmerman, Lukasz Cygan

**Affiliations:** 1 Northwell, New Hyde Park, NY, USA; 2 Staten Island University Hospital, Department of Emergency Medicine, Staten Island, NY; 3 Zucker School of Medicine at Hofstra/Northwell, Hempstead, NY; 4 University of Buffalo, Emergency Medicine Department, Buffalo, NY; 5 New York University Long Island Hospital, Mineola, NY; 6 New York University Grossman Long Island School of Medicine, Mineola, NY; 7 New York-Presbyterian Brooklyn Methodist, Department of Emergency Medicine, Brooklyn, NY

## Abstract

**Audience:**

Emergency medicine residents and medical students

**Introduction:**

Many emergency medicine residency programs have their residents take an in-training exam (ITE) in which performance has been linked to certifying exam pass rates, so solid preparation is important.^1^ The typical clinical schedule of an emergency medicine resident is incredibly varied and time-consuming, which limits time to dedicate to preparation. All emergency medicine programs have synchronous and asynchronous protected time for resident education which provides an excellent opportunity to prepare learners for in-training exams and thus the certifying exam. Gamification is a promising tool some educators have turned to which has been shown to improve attitudes and behaviors towards learning.^2^ This may make it a useful tool for board-review, particularly for the cohort of residents who may have negative attitudes or behaviors towards preparing for it.^3^ This session took inspiration from “The Amazing Race” — a reality television competition where teams race around the world — to provide learners with an exciting activity during dedicated synchronous or asynchronous educational conference time, which may be helpful for board review. Residents may prefer question-based preparation for the in-training exam, so the authors devised The EMazing Race to include some components of this while also adding more clinical practice-relevant topics and activities.^3^

**Educational Objectives:**

By the end of this 2-hour session, learners will demonstrate their knowledge on the following board-related emergency medicine topics:

*Ob/GYN – links to 13.7 Complications of Delivery in Core Model of EM 2022*

1. Know the first maneuver to manage a nuchal knot in an emergency delivery
2. Recognize the signs of shoulder dystocia and learn multiple maneuvers to manage this including McRoberts, suprapubic pressure, Rubin, Woods, and Menticoglou
3. Identify the steps in delivering a fetus in breech positioning and learn about the Gaskin maneuver
4. Understand management of trauma in pregnant patients including maneuvers to manage hypotension, recognition of uterine rupture, placental abruption, as well as rupture of membranes and its diagnostic findings

*Renal/GU – links to 15.0 Renal and Urogenital Disorders in Core Model of EM 2022*

5. Review indications for admission in patients with nephrolithiasis such as intractable nausea and vomiting
6. Recognize signs of acute tubular necrosis by urinalysis findings
7. Recall that staghorn calculi are most commonly composed of magnesium, ammonium, and phosphate
8. Identify appropriate medications to manage hypertension in patients with kidney disease
9. Recall various managements of priapism including intracavernosal irrigation, drainage, and phenylephrine injection
10. List specific indications for emergent hemodialysis including specific medications
11. Identify appropriate medications to manage various GU infections including balanitis and acute cystitis in men and women of varying ages
12. Identify the most common presenting sign of a nephroblastoma

*Splinting – links to 18.1.8.2 Extremity bony trauma, fracture in Core Model of EM 2022*

13. Demonstrate the proper application of the following splinting techniques: thumb spica, sugar tong, and posterior leg splint

**Educational Methods:**

Inspired by the reality TV show competition, “The Amazing Race,” small groups of residents and medical students from all years of training raced through a series of stations separated geographically with distinct educational objectives, guided by clues won after completion of each leg or “task.”

**Research Methods:**

Following completion of the race, participants were provided with a voluntary survey asking them to indicate for the following statements whether they strongly disagree, disagree, are neutral, agree, or strongly agree: (1) The activity was engaging. (2) I prefer this type of learning to traditional lecturing. (3) I found this activity helpful for board/ITE review. (4) I learned something new about Ob/GYN, renal, and/or splinting. (5) Open response: What other feedback do you have? Optional pre- and post-intervention ROSH review questions on OBGYN were administered one month before and one month after the intervention.

**Results:**

Twenty-five out of 28 individuals responded to the survey. All but two individuals stated they “strongly agree” that the activity was engaging. All but one respondent “agree” or “strongly agree” that they prefer this type of learning to traditional lecturing. Sixteen respondents stated “Strongly agree” and four stated “agree” that they found this activity helpful for board/ITE review. Eighteen respondents stated “strongly agree” and seven others stated “agree” that they learned something new about Ob/GYN, renal, and/or splinting. Open-response feedback was overwhelmingly positive and commented on a desire for future similar outdoor educational activities and its benefit as a team-building exercise. The majority of learners (four out of seven) who completed the optional pre- and post- ROSH review questions set on OBGYN content had an increase in their ROSH review score.

**Discussion:**

Gamification has been shown to be a promising tool for improving attitudes and behaviors towards learning, which aligns well with our learner feedback.^1^ Learner feedback was extremely positive, primarily centered around a desire for similar activities in the future. Most learners found the session engaging, preferable to traditional lecturing, and helpful for ITE/board preparation which suggests that the format could be used as an alternative way to review board material. Some studies have shown benefits to outdoor learning, though this has not been examined in emergency medicine didactics specifically.^4^ In the future, the authors intend to employ more gamification in resident education and will trial increased use of outdoor spaces in these activities, given learner preference for this.

**Topics:**

Breech delivery, shoulder dystocia, emergent dialysis, acute kidney injury, trauma in pregnant patients, genitourinary tumors, acute cystitis, nephrolithiasis, balanitis, thumb spica, sugar tong, posterior short leg, splinting, small-group activity, team-building exercise, educational games, gamification, outdoor activities, race.

## USER GUIDE

**Table:** 

List of Resources:	
Abstract	1
User Guide	4
Small Groups Learning Materials	8
Appendix A: Small Group Application Exercise	8
Appendix B: Quiz Questions	14
Appendix C: Quiz Answers	30
Appendix D: Graphics	46
Appendix E: Welcome PowerPoint	49

**Learner Audience:**

Medical Students, Interns, Junior Residents, Senior Residents

**Time Required for Implementation:**

The lead facilitator should expect to spend approximately five hours for planning/set-up. For the remaining facilitators, they should expect a three-hour time commitment to participate in this activity from set-up to breakdown. The activity itself lasted two hours for this group but could be shorter or longer depending on how many learners a program has and which activities are included.

**Topics:**

Breech delivery, shoulder dystocia, emergent dialysis, acute kidney injury, trauma in pregnant patients, genitourinary tumors, acute cystitis, nephrolithiasis, balanitis, thumb spica, sugar tong, posterior short leg splinting, small-group activity, team-building exercise, educational games, gamification, outdoor activities, race.

**Objectives:**

By the end of this 2-hour session, learners will demonstrate their knowledge on the following board-related emergency medicine topics:

*Ob/GYN – links to 13.7 Complications of Delivery in Core Model of EM 2022*

1. Know the first maneuver to manage a nuchal knot in an emergency delivery
2. Recognize the signs of shoulder dystocia and learn multiple maneuvers to manage this including McRoberts, suprapubic pressure, Rubin, Woods, and Menticoglou
3. Identify the steps in delivering a fetus in breech positioning and learn about the Gaskin maneuver
4. Understand management of trauma in pregnant patients including maneuvers to manage hypotension, recognition of uterine rupture, placental abruption, as well as rupture of membranes and its diagnostic findings

*Renal/GU – links to 15.0 Renal and Urogenital Disorders in Core Model of EM 2022*

5. Review indications for admission in patients with nephrolithiasis such as intractable nausea and vomiting
6. Recognize signs of acute tubular necrosis by urinalysis findings
7. Recall that staghorn calculi are most commonly composed of magnesium, ammonium, and phosphate
8. Identify appropriate medications to manage hypertension in patients with kidney disease
9. Recall various managements of priapism including intracavernosal irrigation, drainage, and phenylephrine injection
10. List specific indications for emergent hemodialysis including specific medications
11. Identify appropriate medications to manage various GU infections including balanitis and acute cystitis in men and women of varying ages
12. Identify the most common presenting sign of a nephroblastoma

*Splinting – links to 18.1.8.2 Extremity bony trauma, fracture in Core Model of EM 2022*

13. Demonstrate the proper application of the following splinting techniques: thumb spica, sugar tong, and posterior leg splint

### Linked objectives and methods

Objectives 1–12 are accomplished through completion and faculty/group review of the four quizzes in the “Detour” leg of the race, which has various multiple choice, true/false, and matching questions to illustrate each objective. These learning points are further solidified by the review of the “Pearls” for this educational session. Objective 13 is accomplished in the “Roadblock” leg of the race where contestants race to correctly complete the splints as evaluated by faculty. Objective 14 is evident throughout the activity which requires small groups to work together to complete a series of difficult tasks.

### Recommended pre-reading for facilitator

- Consider watching episodes of “The Amazing Race” to become familiar with the format. Review all materials provided here including answers to quiz questions.

### Learner responsible content (LRC)

- Although not required, the authors’ program assigned Rosh Review questions for Ob/GYN and Renal/GU approximately 1 month pre- and post-intervention.

### Small group application exercise (sGAE)

See the following attached materials for this small group exercise

- Appendix A: sGAE
- Appendix B: Quiz Questions
- Appendix C: Quiz Answers
- Appendix D: Graphics (Route Info, Roadblock, Detour)
- Appendix E: Welcome PowerPoint

### Results and tips for successful implementation

This game was tested on a total of 28 learners in the fall of 2023, which consisted of emergency medicine residents and two rotating medical students. Twenty-five out of 28 individuals responded to a post-intervention survey.

All but two individuals stated they “strongly agree” that the activity was engaging; the remaining people stated “strongly disagree” or “neutral.” All but one respondent “agree” or “strongly agree” that they prefer this type of learning to traditional lecturing; the last selected “neutral.” Sixteen respondents stated “Strongly agree” and “agree,” four selected “neutral,” and one stated “disagree” that they found this activity helpful for board/ITE review. Eighteen respondents stated “strongly agree” and seven others stated “agree” that they learned something new about Ob/Gyn, renal, and/or splinting.

To the open response question, learners stated:

“This was awesome,” and “more conferences outside!”

“Let’s do more outdoor sections of conference. The half inside half outside is great!”

“More outdoor events.”

“More utilization of nice weather and the park is always welcome as a substitute for traditional lecture style learning.”

“Great for team building and camaraderie.”

“Great interactive way to make learning fun! Was also great for team building. Would absolutely recommend doing it again and making it an annual event.”

Two respondents requested “Easier directions,” and a last person suggested “Less distance between stations might be good if you want to include more stations for more learning opportunities.”

Residents were provided with optional pre- and post-intervention OBGYN and renal/GU ROSH review questions. Seven residents completed both pre- and post-intervention OBGYN questions. None completed the renal/GU questions. Of those residents, most (4 out of 7) had an increase in their ROSH review score 1-month post-intervention. There was no correlation between the winners of the race and those who completed the review questions, though the winning team members all had an increase in their ITE scores for OBGYN and renal/GU topics when compared to pre-intervention. For the remaining participants, ITE scores were compared pre- and post-intervention and for OBGYN sub-scores, four out of 15 increased their score, and seven out of 15 residents maintained their score post-intervention. For renal/GU sub-scores, five out of 15 increased their score, and four out of 15 maintained their score post-intervention. While none of these increases were statistically significant, they at least indicate equivalency to some degree, and when combined with the qualitative data showing learner preference compared to traditional teaching techniques, suggest value for this gamified session.

Of note, two medical students were involved in the activity and ultimately matched at the authors’ program, which may indicate that showcasing unique conference activities during interview/audition season holds recruitment value for programs.

Regarding implementation, the authors have several suggestions to fit the needs of the readers’ programs.

- Quiz content can easily be switched out depending on where in their curriculum one’s program is.
- Delays such as purchasing an item from a restaurant could be changed to non-monetary options like running around the block or timing out a delay on a stopwatch.
- The puzzles in the “Detour” leg or the lawn games in the “Roadblock” leg could also be switched out to other games or eliminated depending on what a program has available.
- Most of the authors’ residents commute to work by foot, bicycle, or mass transportation but the race could include driving between legs if more applicable to the reader.
- While no training was provided for small group dynamics prior to this exercise, the authors advise strong consideration of this.
- The authors purchased “Amazing Race” tearaway envelopes on Amazon.com, but simple letter envelopes could also be used.
- The authors purchased children’s puzzles on Amazon.com, specifically, “eeBoo: Ready to Learn: Human Anatomy 4-Puzzles - Body Systems Set of 4–48 Piece Jigsaws, Includes Educational Poster, Kids Ages 8+,” but any similar puzzle will suffice.
- The specific route info clues were not included in this manuscript because they are specific to the authors’ geographic location, but readers should devise their own as it fits into their race design.
- This is a time and facilitator intensive intervention. Consider shortening the number of stations or games to best fit your program needs.
- While the race format increased engagement and enjoyment, the authors recognize that time constraints and activity pacing may limit the depth and breadth of content covered. The authors recommend selecting focused, high-yield topics for inclusion and using the end-of-race debrief to address nuances or expand on complex subject matter. This activity is intended to cover high-yield topics for board review and clinical practice, not to replace primary learning through independent reading, structured didactics, or the core conference curriculum.
- Programs with limited space, staff, or weather flexibility can fully replicate the EMazing Race indoors. Consider using adjacent hospital classrooms, simulation labs, or even different corners of a classroom. For example, conference rooms can serve as the Detour quiz stations, a simulation bay can be repurposed for the splinting Roadblock, and a hallway or large room can be adapted for physical mini-challenges such as beanbag toss or chair races. Route clues can direct to specific stations by name or continue to use riddles leading to different rooms in the hospital, and timing challenges can replace travel delays.

### Pearls

#### Ob/GYN

- Pearl #1: The first step when a nuchal cord is present in an emergency delivery is to reduce the cord by slipping it over the fetal head and proceed with delivery. If the cord is wound too tightly or the above maneuver fails, the cord can be clamped in two places and cut to proceed.
- Pearl #2: Shoulder dystocia may first be recognized by failure to deliver anterior shoulder and retraction of fetal head against the perineum (“turtling”). There are multiple maneuvers to manage this obstetric emergency, but the practitioner should begin with McRoberts and simultaneous suprapubic pressure, whereby the mother’s legs are sharply flexed up to the abdomen and held in place. Additional maneuvers include Rubin and Woods, which involve rotating the posterior shoulder either towards fetal face or towards fetal back respectively and rotating fetus 180 degrees to dislodge trapped shoulder. Another option is the Menticoglou maneuver which delivers the posterior shoulder first.
- Pearl #3: For breech deliveries, the practitioner should allow the delivery to proceed as naturally as possible with little traction or force applied. Legs typically deliver spontaneously and should be supported by the practitioner by holding both legs at popliteal fossa. Ideally, the sacrum of the fetus should be anterior and can be supported by the practitioner with thumbs on sacrum and hands on fetal pelvis. When the head is ready to deliver, the practitioner should place fingers on the fetal maxilla, maintain cervical flexion, and support delivery by rotating the fetus up and out towards the mother. Additionally, various maneuvers exist to aid in the breech delivery, including the Gaskin maneuver where patient is placed in all-fours.
- Pearl #4: For hypotensive pregnant patients, the first maneuver is placing the patient in the left lateral decubitus position to relieve pressure on the inferior vena cava. Pregnant patients who have experienced blunt trauma are at risk of a variety of complications. The most sensitive sign of placental abruption is uterine irritability. Uterine rupture would be signified by loss of uterine contour. Rupture of membranes can be diagnosed by ferning on microscopic evaluation, pH of 7 of the fluid, presence of fetal fibronectin. All pregnant trauma patients over 20 weeks gestation should undergo 4 hours of fetal monitoring, and Rh-negative patients should receive Rhogam.

#### Renal/GU

- Pearl #5: Regardless of kidney stone size, intractable nausea and vomiting is an indication for admission. Other ED indications include solitary kidney, renal failure, obstructing stone with infection, or uncontrolled pain.
- Pearl #6: Acute tubular necrosis will have urinalysis findings of brown granular casts.
- Pearl #7: Staghorn calculi are most commonly composed of magnesium, ammonium, and phosphate (struvite stones), and are often associated with urease-producing organisms like *Proteus*.
- Pearl #8: In treatment of hypertension in patients with polycystic kidney disease, the preferred initial agent is an angiotensin-converting enzyme inhibitor.
- Pearl #9: Priapism can be managed with intracavernosal irrigation, drainage, and phenylephrine. Ischemic (low-flow) priapism is painful, rigid, and lasts >4 hours; it is a urologic emergency. Risk factors include sickle cell disease, trazodone, antipsychotics, and intracavernosal injections. Cavernoma blood gas will show hypoxia and acidosis. High-flow (non-ischemic) priapism is usually painless and typically follows trauma.
- Pearl #10: The diagnosis of spontaneous bacterial peritonitis in patients on peritoneal dialysis is different from those on conventional hemodialysis. 100 WBC/mcL or > 250 PMN/mm^3^ is diagnostic of this condition.
- Pearl #11: The mnemonic “AEIOU” can be used to recall emergent indications for hemodialysis. This stands for acidosis, electrolyte abnormalities, ingestion, overload, and uremia. For managing hypercalcemia electrolyte abnormalities, a calcium level >18 mg/dL, presence of renal failure, heart failure, or neurologic symptoms are all indications for emergent dialysis. Only certain ingestions in overdose are dialyzable. Various mnemonics exist to aid in remembering these; one such is “PLASMA TV,” which stands for Phenobarbital, Lithium, Acidosis, Salicylates, Metformin, Alcohols, Theophylline, Valproic Acid. Not included in this list is carbamazepine.
- Pearl #12: There are various genitourinary infections that can occur in patients. Balanitis is treated with topical antifungals and may be the first presenting sign of diabetes. Urinary tract infection treatment in males differs depending on age and risk factors–young and sexually active should receive coverage for sexually transmitted infections, whereas older men or men who have sex with men (MSM) generally should receive coverage for enteric bacteria. For women, medications like ceftriaxone, fluoroquinolones, and cefpodoxime are generally reserved for complicated cystitis.
- Pearl #13: The most common presenting sign of a nephroblastoma is a painless abdominal mass. It typically occurs in children under 5 and requires prompt imaging and urologic referral.

**Figure:**
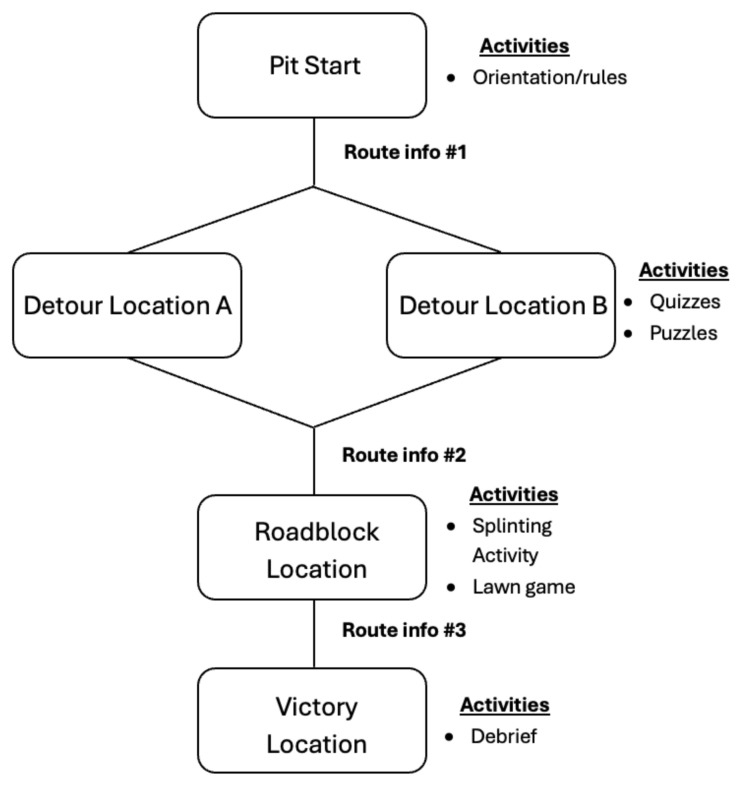
Basic race layout with associated activities

## Supplementary Information



## Figures and Tables

**Image 1:**
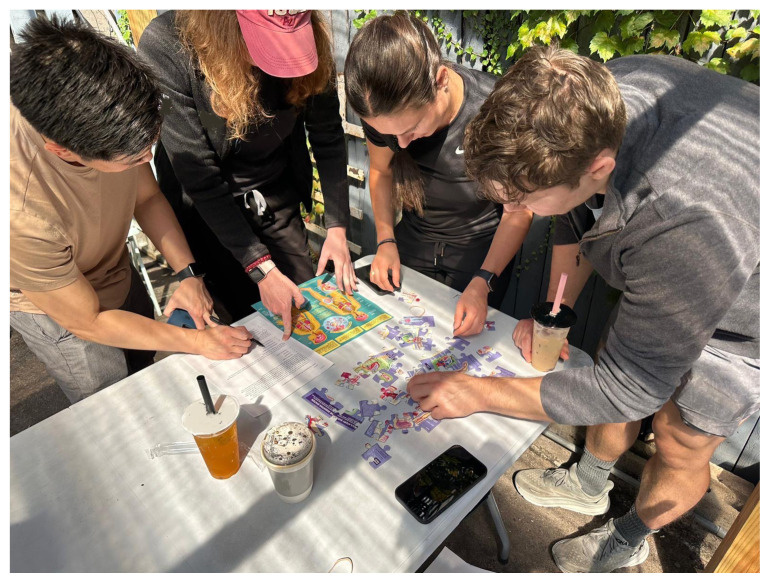
A team completing the “Detour” challenge including a puzzle, quizzes, and their purchased items as time-delays. Authors’ own image. Brendan Freeman. Untitled image. In: Freeman B. *Personal Collection* [online image]. Unpublished; 2024

**Image 2:**
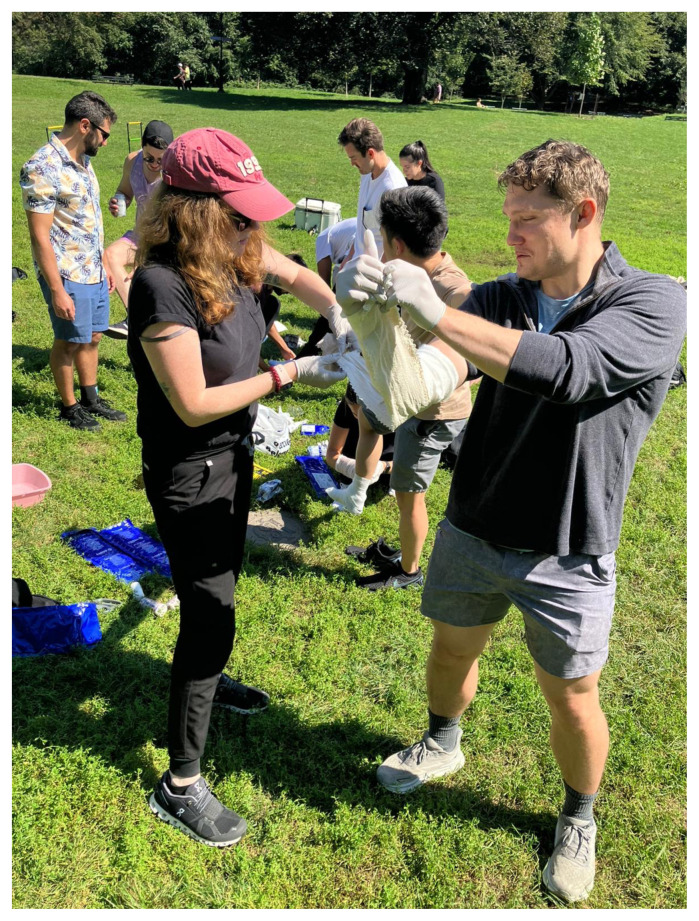
a team completing the splinting challenge in the “roadblock” leg. Authors’ own image. Brendan Freeman. Untitled image. In: Freeman B. *Personal Collection* [online image]. Unpublished; 2024

**Figure:**
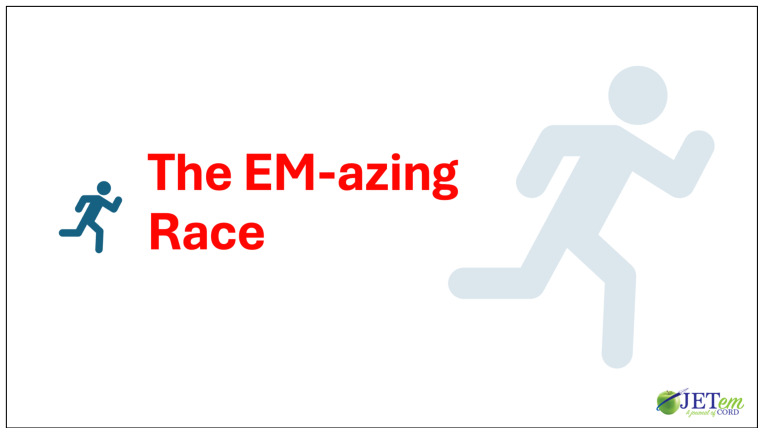
Please see associated PowerPoint file

## SMALL GROUPS LEARNING MATERIALS

### Appendix A Small Group Application Exercise

#### Basic Rules

The race is divided into several legs; the first begins at a starting area called the “Pit Start.” At the Pit Start all groups receive an envelope containing a “Route Info Clue” directing them to one of two Detour Locations (the next leg). At each Detour Location, the teams receive another envelope containing “Detour Tasks” -- two tasks that must be completed as a group. Successful completion provides them with an envelope containing another “Route Info Clue” which leads them to the next leg, known as the Roadblock location. At that leg, groups receive a new envelope containing a “Roadblock Task.” The Roadblock directs them to select one member of the team to complete a task while the rest of the team completes a different task. Successful completion of both tasks provides them with a final envelope containing a “Route Info clue” leading to the last location and victory.

#### Pre-Activity Setup

1. **Recruit Facilitators:**
  ○ Assign facilitators to each leg, ideally two facilitators for each leg and one main-lead facilitator to oversee the entire event.
2. **Group Assignments:**
  ○ Before the event, obtain a list of all learners participating. Divide them into groups of four to five individuals, ensuring an even mix of medical students and residents from every year of training.
3. **. Communication Setup:**
  ○ Create a group chat using a service such as WhatsApp that includes all facilitators to ensure seamless communication throughout the event.
4. **Select Station Locations:**
  ○ **Pit Start (Leg I):** Choose a starting location large enough to hold all facilitators and learners. The authors used the resident conference room at the hospital.
  ○ **Detour Locations (Leg II):** Choose two locations that can accommodate approximately 20 participants (faculty and learners). For example, local businesses or restaurants. Ensure that the two locations are equidistant from the “Pit Start” to ensure race fairness. The location should have tables and chairs for quiz administration, and the property owners should approve of the activity in advance so as not to disrupt their daily operations. The authors selected two local restaurant partners with outdoor seating. If selecting local restaurants, the authors advise reserving enough tables for two hours at each location and telling the businesses that purchases will be made as a part of the activity.
  ○ **Roadblock Location (Leg III):** Select a large outdoor space, such as a local park, that can accommodate all participants, lawn games, and splinting activities.
  ○ **Tip for selecting Leg Locations:** Ensure that all leg locations are familiar to participants or easily findable on services like Google Maps so that clues are solvable.
5. **Print and Assemble Materials:**
  ○ Prepare and print all necessary materials and assemble envelopes for each team.
    1. A “Detour” envelope for every team (half with Detour Location A, half with Detour Location B) (see Appendix C)
    2. Three “Route Info” envelopes for every team (Appendix C)
    3. A “Roadblock” envelope for every team (Appendix C)
    4. Quizzes and answers (Appendix A and B)
  ○ Label the envelopes for clarity and ease of distribution
6. **Purchase any supplemental materials**
  ○ This includes purchasing or borrowing any needed lawn games, puzzles, and branded envelopes

#### Pre-Briefing

1. **Confirm Participation:**
  ○ If you are using restaurant community partners, the lead facilitator should contact them on the day of the event to confirm their participation.
2. **Facilitator Meeting:**
  ○ One hour before the event starts, all facilitators should meet at the “Pit Start” location to review the rules and expectations. During this meeting, facilitators should also receive all necessary materials for their stations.
3. **Station setup:** send the facilitators to set-up the remainder of each of their stations. For specifics, see below.

#### Stations

##### Materials/preparation

1. PowerPoint detailing race overview; see Appendix D
2. tear-away envelope for each team containing a “Route Info Clue;” see Appendix C

##### Instructions

Welcome all participants and review the rules and layout of the race using the attached PowerPoint (Appendix D). Leave time for questions and then give each team a tear-away sealed envelope containing the first Route Info Clue. Once all teams receive the envelope, the race begins.

#### Leg II: “Detour”

##### Materials

1. 48-piece children’s anatomy puzzles. You will need as many puzzles as there are groups of learners in your race. The pieces of each puzzle should be split into four baggies containing 12 pieces each.
2. A tear-away envelope for each group of learners containing “Route Info”; see Appendix C for graphics
3. Four quizzes for each group of learners (authors advise printing multiple copies of each); see Appendix A
4. Printed copy of the quiz answers for facilitator; see Appendix B

##### Instructions

Facilitators should arrive before the start of the race to set-up each section and to open a tab (if applicable) at their designated Detour location. Each table should have a 48-piece puzzle with pieces split into four baggies. As each team arrives, they are provided with quiz #1. Each team will work together to complete four sequential multiple-choice quizzes on Ob/GYN and renal/GU topics. Following completion of each quiz, the facilitator will grade it using the answer sheet and, if the target score is reached, team members are given one of the baggies containing twelve pieces of the anatomy puzzle and the next quiz. If the target score is not reached, then one team member must purchase an item from the Detour Location on the open tab. The team must fix their incorrect answers on the quiz (participants may look-up answers electronically at this point). Progression to the next quiz cannot start until the team member is seated with the purchased item and a passing score is achieved on that quiz. There are a total of four quizzes and four baggies of puzzles given between each successful completion of a quiz. To move on to the next leg, teams must complete all four quizzes and complete the 48-piece anatomy puzzle. Teams ready to progress are provided with another tear-away envelope provided by the faculty at that station containing a “Route Info Clue”

#### Leg III: “Roadblock”

##### Materials

1. tear-away envelope for each group containing a “Roadblock” task
2. “Chutes” Game:
  - 2 ~five-foot-tall poles/stakes (eg, ski poles)
  - 2 empty glass bottles
  - 2 frisbees
3. “Ladders” Game:
  - 1 ladder toss game set (a.k.a. “Ladder Ball”) containing 2 wooden ladder frames and 6 bolas balls.
4. Splinting material including stockinette, Webril, orthoglass or plaster rolls, ace bandages, tape, bins of water to harden, trauma shears, nitrile gloves

##### Instructions

Facilitators should arrive prior to the start of the race to set up the stations. They should designate an area for each lawn game and an area for splinting. The stakes are placed into the ground next to each other and a glass bottle is balanced on top of each. Frisbees should be placed on the ground approximately 10 feet away from the stakes. Ladder toss is set up similarly with two of the ladders placed next to each other and a set of bolas placed on the ground approximately 10 feet away. When teams arrive at this location, they are met by the faculty facilitators and are given another tear-away envelope containing the “Roadblock” task. As defined in the basic rules, a Roadblock entails that one team member must complete a task alone. The team can decide which team member will do the solo task, and that team member may pick between two lawn games: “Chutes” or “Ladders.” In the game of “Chutes,” the player throws a frisbee at a bottle balanced on a stick and knocks it off to score a point. In the game of “Ladders,” the player throws a bola at a ladder with three rungs and must wrap the bola around any rung to score a point. While that team member is playing their selected lawn game, the remaining team members are participating in a splinting race. The facilitator assigns each teammate a splint by providing each team member with a fracture type, such as a thumb spica for scaphoid fracture, a sugar tong for Colles fracture, and a short leg posterior splint for a distal fibula fracture. There should be one type of splint per person. Completion of this leg is marked by approval of all splints by the facilitator and the team member hitting the target on either lawn game a total of four times.

#### Leg IV: “Finish Line”

##### Materials

1. tear-away envelope for each group containing a “Route Info;” see Appendix C for graphics

##### Instructions

Teams who complete all the above legs successfully should be provided with a last tearaway envelope which contains a “Route Info Clue” directing participants to the finish line. When all teams finish the race, consider regrouping at finish line or pit start to review the provided “Pearls” worksheet.

### Appendix B Quiz Questions

#### Quiz 1

*Instructions: A passing score for this section is 5 out of the 7 questions correct. The faculty facilitator will review all answers with your team. If the target score is achieved, you will be given a section of a puzzle to complete and the next quiz. If the target score is not reached, your team will have to purchase an item on the department tab from the store. The next puzzle section and quiz will only be given when the entire team is seated with the purchased item, and you have corrected your answers (you may electronically look up answers if it’s your second attempt).*

1. A 24-year-old woman at approximately 40-weeks’ gestation arrives at the emergency department in active labor with the fetal head visible at the introitus with a nuchal cord. Which of the following actions should be prioritized?
  - Gently reduce the cord over fetal head and proceed with delivery process
  - Administer medication to accelerate the delivery process
  - Secure the cord in two places with clamps and cut between the clamps
  - Elevate the presenting fetal part and proceed to emergency cesarean section
2. A 23-year-old G3P2 woman with a history of polyhydramnios presents to the emergency department with a precipitous delivery. The fetal head delivers easily, but then the head retracts tightly against the perineum. Which of the following is the next most appropriate step?
  - Flex the mother's thighs sharply against her abdomen to widen the pelvic outlet and apply suprapubic pressure
  - Insert a finger into vagina and hook around the inferior clavicle, applying caudal pressure until it fractures
  - Rotate the fetus’ anterior shoulder towards the back to disengage it from behind the maternal pubic bone
  - Attempt to rotate the posterior shoulder into the anterior-posterior diameter of the pelvis

For the following questions, match the name of the appropriate maneuver to its picture and description.

3. The Wood’s Maneuver
4. The McRoberts Maneuver
5. The Menticoglou Maneuver
6. The Gaskin Maneuver
7. The Rubin Maneuver

Image 1:

The birthing person is positioned on hands and knees, leaning forward onto their forearms or hands, and their knees are spread apart. This posture allows gravity to aid the descent of the baby through the birth canal, can widen pelvic outlet, and reduce pressure on perineum.

Answer: _______________

Image 2:

The birthing person is positioned flat on their back, with legs sharply flexed at the hips and pushed back toward shoulders. This positioning helps to widen the pelvic outlet by rotating the pelvis, which can sometimes free the impacted shoulder and allow the baby to be delivered. Providers may combine this maneuver with other techniques, such as suprapubic pressure to further facilitate the baby's delivery.

Answer: _______________

Image 3:

The healthcare provider places one hand on the posterior or anterior shoulder (whichever is more accessible) and applies pressure **to adduct the shoulders together**. The baby is then rotated 180 degrees to help deliver the posterior shoulder anteriorly.

Answer: _______________

Image 4:

The healthcare provider rotates the posterior shoulder **counterclockwise** until it is delivered anteriorly.

Answer: _______________

Image 5:

The birthing person is usually lying on back in a standard delivery position. The provider hooks both of their middle fingers under the axilla of the posterior arm. Another provider gently flexes the head of the fetus towards the anterior shoulder. Gentle traction is used to guide the posterior shoulder along the curvature of the sacrum until the posterior shoulder is delivered.

Answer: _______________

#### Quiz 2

*Instructions: A passing score for this section is 6 out of the 7 questions correct. The faculty facilitator will review all answers with your team. If the target score is achieved, you will be given a section of a puzzle to complete and the next quiz. If the target score is not reached, your team will have to purchase an item on the department tab from the store. The next puzzle section and quiz will only be given when the entire team is seated with the purchased item, and you have corrected your answers (you may electronically look up answers if it’s your second attempt).*

The following questions pertain to a **breech** presentation if cesarean is not immediately available:

1. The fetal sacrum should be in which position for an ideal vaginal delivery in breech presentation?
  - Anterior
  - Posterior
  - Oblique
  - Right sacrum anterior
  - Left sacrum anterior
2. TRUE OR FALSE? Traction should be placed onto fetal buttocks to deliver umbilicus
  - TRUE
  - FALSE
3. TRUE OR FALSE? Fetal legs usually deliver spontaneously
  - TRUE
  - FALSE
4. Which of the following is the optimal practitioner hand positioning for **delivery of fetal legs** in breech presentation?
  - Grasp both legs at ankles and deliver
  - Grasp one leg at ankle, deliver, then repeat for other leg
  - Grasp both legs at popliteal fossa and deliver
  - Grasp one leg at popliteal fossa, deliver, then repeat for other leg
5. When **shoulders** are being delivered in breech presentation, which of the following is the optimal practitioner hand/finger placement on fetus?
  - Thumbs on abdomen, hands on back
  - Thumbs on back, hands on abdomen
  - Thumbs on sacrum, hands on pelvis
  - Thumbs on sacrum, hands on abdomen
  - Thumbs on shoulders, hands on chest
6. When the **fetal head** is being delivered in breech presentation, which of the following describes the correct technique?
  - Maintain cervical FLEXION by placing one hand on the fetal occiput and shoulders, apply flexing pressure on the occiput, and place the fingers of the other hand on the infant’s MANDIBLE to aid in cervical flexion.
  - Maintain cervical FLEXION by placing one hand on the fetal occiput and shoulders, apply flexing pressure on the occiput, and place the fingers of the other hand on the infant’s MAXILLAE to aid in cervical flexion.
  - Maintain cervical EXTENSION by placing one hand on the fetal occiput and shoulders, apply flexing pressure on the occiput, and place the fingers of the other hand on the infant’s MANDIBLE to aid in cervical flexion.
  - Maintain cervical EXTENSION by placing one hand on the fetal occiput and shoulders, apply flexing pressure on the occiput, and place the fingers of the other hand on the infant’s MAXILLA to aid in cervical flexion.
7. To complete delivery, which is the correct direction to rotate fetal legs?
  - Rotate fetal legs up and towards mother
  - Rotate fetal legs down and towards the ground

#### Quiz 3

*Instructions: A passing score for this section is 8 out of the 10 questions correct. The faculty facilitator will review all answers with your team. If the target score is achieved, you will be given a section of a puzzle to complete and the next quiz. If the target score is not reached, your team will have to purchase an item on the department tab from the store. The next puzzle section and quiz will only be given when the entire team is seated with the purchased item, and you have corrected your answers (you may electronically look up answers if it’s your second attempt).*

1. Which of the following patients with renal colic meets the criteria for hospital admission based on their clinical presentation?
  - A 40-year-old woman with a 3 mm stone at the ureteropelvic junction, complaining of moderate flank pain that is partially relieved by analgesics, with no signs of infection or dehydration.
  - A 35-year-old man with a 6 mm stone at the renal pelvis, having intermittent colicky pain that responds well to oral pain medication, no hydronephrosis on ultrasound, and presenting with normal vital signs and urinalysis results.
  - A 28-year-old pregnant woman with a 4 mm stone at the ureterovesicular junction, reporting mild to moderate pain, normal vital signs, and a urinalysis showing 1+ blood.
  - A 30-year-old man with a 5 mm stone at the ureterovesicular junction, experiencing uncontrolled pain despite intravenous pain medication.
2. Which of the following microscopic findings is most characteristic of acute tubular necrosis (ATN) in renal biopsy specimens?
  - Hyaline cell casts
  - Presence of granular or muddy brown casts in the tubular lumens
  - Proliferation of inflammatory cells and fibroblasts within the interstitium
  - Diffuse thickening of the glomerular basement membrane with immune complex deposits
3. A 55-year-old male patient presents to the emergency department with severe left-sided flank pain, hematuria, and dysuria for the past 24 hours. He has a history of recurrent urinary tract infections and kidney stones. On physical examination, he appears uncomfortable, and his blood pressure is 140/90 mmHg. Laboratory tests reveal leukocytosis and microscopic hematuria. A non-contrast CT scan of the abdomen and pelvis is performed, showing a large, branching stone that fills the renal pelvis and extends into the calyces. Which of the following compositions is most likely responsible for the formation of the calculus described in this patient's CT scan?
  - Calcium Oxalate
  - Uric Acid
  - Magnesium, Ammonium, and Phosphate
  - Cystine
4. A 50-year-old woman presents to her primary care physician for a routine check-up. She has a family history of kidney disease and hypertension. On examination, her blood pressure is 150/92 mmHg. Laboratory tests reveal elevated serum creatinine and proteinuria. Renal ultrasound shows enlarged kidneys with multiple cysts bilaterally. Which of the following blood pressure medications is the most appropriate initial choice to start in this patient?
  - Angiotensin-Converting Enzyme Inhibitor
  - Beta-Blocker
  - Calcium Channel Blocker
  - Thiazide diuretic
5. A 34-year-old man with polysubstance use disorder presents to the emergency department complaining of a painful, prolonged erection that has lasted for four hours. He denies any recent sexual activity or trauma. On examination, his penis is erect and tender. The rest of the physical examination is unremarkable. His vital signs are stable. What is the most appropriate initial management for this patient's condition?
  - Intracavernosal injection of alpha-adrenergic agonist
  - Immediate consultation with urology for surgical exploration
  - Oral phosphodiesterase-5 (PDE-5) inhibitor
  - Ice packs to the perineum and scrotum
6. A 58-year-old man with end-stage renal disease presents to the emergency department with abdominal pain, fever, and cloudy peritoneal dialysate fluid. On examination, he is febrile, and abdominal tenderness is noted. Peritoneal fluid analysis is performed via paracentesis. Which of the following findings on peritoneal fluid analysis is most consistent with the diagnosis of spontaneous bacterial peritonitis in this patient?
  - Peritoneal fluid polymorphonuclear (PMN) cell count >250 cells/mm^3^
  - Peritoneal fluid glucose of <50 mg/dL
  - Peritoneal fluid culture yielding normal skin flora
  - Peritoneal fluid demonstrating LDH levels ⅔ the upper limit of normal
7. A 45-year-old man with a history of bipolar disorder and chronic kidney disease stage 5, secondary to polycystic kidney disease, presents to the emergency department after a suicide attempt by ingesting multiple medications. He is agitated, tremulous, and hypertensive. The toxicology screen reveals the presence of several drugs in his system. If ingested by the patient, which of the following drugs could be removed by hemodialysis?
  - Digoxin
  - Lorazepam
  - Carbamazepine
  - Metoprolol
8. A 28-year-old pregnant woman at 36-weeks’ gestation is brought to the emergency department after a motor vehicle accident. She complains of abdominal pain and contractions. On examination, she appears distressed, and her vital signs are stable. Abdominal palpation reveals tenderness and guarding. During the speculum examination, you notice bright red blood coming from the cervical os. Which of the following physical exam findings is most indicative of uterine rupture following trauma in this patient?
  - Uterine tenderness to palpation
  - Bright red blood from the cervical os
  - Loss of palpable uterine contour
  - Decreased fetal movement and heart rate
9. A 26-year-old pregnant woman at 38-weeks’ gestation is brought to the labor and delivery unit due to leakage of fluid from her vagina. She denies any recent trauma or discomfort. On examination, there is a pool of clear fluid in the vaginal vault. The patient reports that she has been feeling continuous leakage for the past hour. Which of the following characteristics of the fluid is most indicative of presence of amniotic fluid in this patient?
  - Yellow-brown color
  - Fishy odor
  - Acidic pH
  - Presence of ferning pattern under a microscope
10. A 30-year-old pregnant woman at 34-weeks’ gestation presents to the emergency department following a motor vehicle accident. She was a restrained driver and was involved in a frontal collision. She is experiencing abdominal discomfort. On examination, she appears anxious, her vital signs are stable, and there are no visible external injuries. What is the most sensitive clinical finding that raises suspicion for placental abruption in this patient?
  - Visible vaginal bleeding
  - Fetal bradycardia on Doppler
  - Uterine irritability
  - Maternal tachycardia

#### Quiz 4

*Instructions: A passing score for this section is 8 out of the 10 questions correct. The faculty facilitator will review all answers with your team. If the target score is achieved, you will be given the final section of a puzzle to complete. If the target score is not reached, your team will have to purchase an item on the department tab from the store. The final puzzle section will only be given when the entire team is seated with the purchased item, and you have corrected your answers (you may electronically look up answers if it’s your second attempt).*

1. A 65-year-old man with newly diagnosed end-stage renal disease (ESRD) is brought to the emergency department after a hemodialysis session. He underwent an extended hemodialysis session due to significant fluid overload. Shortly after dialysis, he developed a severe headache, nausea, vomiting, and confusion. His blood pressure is 180/100 mmHg, and he appears restless and disoriented. Which of the following medications is most appropriate to initiate in this patient?
  - Lorazepam
  - Mannitol
  - Furosemide
  - Acetaminophen
2. A 4-year-old child is brought to the pediatrician due to parental concern about a swelling in the abdomen. On examination, the physician palpates a firm mass in the left upper quadrant of the abdomen that does not cross the midline. The child's parents report that the mass has been gradually increasing in size over the past few weeks. The child has no history of trauma or recent illnesses, and there are no other notable symptoms. What is the most common presenting sign of this condition?
  - Hematuria
  - Abdominal pain
  - Painless abdominal mass
  - Fever and weight loss
3. An 86-year-old uncircumcised man presents to the emergency department from a nursing home for complaints of redness, swelling, and discomfort around the head of his penis. He denies any history of sexually transmitted infections and reports that he has trouble keeping his genitourinary area clean. On examination, there is erythema, edema, and discharge accumulating beneath the foreskin. The rest of the genital examination is unremarkable. His vital signs are normal. What is the most appropriate initial treatment for this patient's condition?
  - Topical corticosteroids
  - Oral antibiotics
  - Topical antifungal cream
  - Warm saline baths and gentle retraction of the foreskin
4. A 28-year-old woman presents to the clinic with dysuria, urgency, and increased frequency of urination for the past two days. She denies any fever or back pain. Urinalysis reveals the presence of leukocytes and nitrites. The patient has no known allergies and is not pregnant. Assuming no specific local resistance patterns, what is the most appropriate initial treatment for this patient's condition?
  - Oral fluoroquinolone antibiotics
  - Oral cefpodoxime
  - Oral nitrofurantoin
  - Intravenous ceftriaxone
5. TRUE OR FALSE? Ionizing radiation should be withheld during trauma assessment in pregnant patients to protect the fetus
  - TRUE
  - FALSE
6. A 28-year-old pregnant woman at 35-weeks’ gestation is admitted to the emergency department with symptoms of dizziness, lightheadedness, and hypotension (blood pressure 90/60 mmHg). She is conscious, but her skin appears pale and clammy. Fetal heart rate is within the normal range. The obstetrician is called for consultation. What is the most appropriate initial patient positioning to address hypotension in this pregnant patient?
  - Supine position with a wedge under the right hip
  - Left lateral tilt
  - Sitting upright
  - Trendelenburg position
7. A transvaginal ultrasound is performed on a patient pregnant at 34 weeks who presented to the ED complaining of abdominal pain after a low-speed motor vehicle collision. Her uterus is tender to palpation. An ultrasound was obtained which demonstrates a focal hypoechoic area behind the placenta and signs of fetal distress. This presentation is most suggestive of which of the following pathologies?
  - Uterine rupture
  - Intrauterine fetal demise
  - Placental abruption
  - Placenta previa
8. A 45-year-old man presents to the urology clinic with sudden-onset pain and swelling in his right testicle. He reports dysuria and a frequent urge to urinate. On examination, there is tenderness and warmth over the right epididymis. He is sexually active with other males. The patient's medical history is notable for a recent urinary tract infection. Assuming no specific local resistance patterns, what is the most appropriate initial treatment for this middle-aged man with symptoms suggestive of epididymitis?
  - Oral levofloxacin and IM ceftriaxone
  - Oral levofloxacin
  - IM ceftriaxone and oral doxycycline
  - Immediate referral for surgical exploration and drainage
9. A 30-year-old man with sickle cell disease presents to the emergency department with a painful, persistent erection lasting for 4 hours. Physical examination reveals a rigid, non-tender penis with no signs of injury. Initial attempts at conservative management, including ice packs and hydration, have failed to resolve the priapism. What is the most appropriate next step in managing this patient's priapism?
  - Intravenous administration of phenylephrine
  - Oral administration of pseudoephedrine
  - Aspiration of corpus cavernosum
  - Emergent dorsal slit procedure
10. A 60-year-old female with a history of multiple myeloma presents to the emergency department with nausea, vomiting, and severe joint pains. Her vital signs are notable for a blood pressure 190/110 mmHg, and laboratory results show a pH 7.20, serum potassium 6.2 mEq/L, BUN of 64, and a creatinine of 3.4. Her calcium level is 14. Which of the following is an indication for emergent dialysis?
  - Metabolic acidosis with a pH <7.20
  - Hyperkalemia >6 mEq/L
  - Altered mental status
  - Presence of renal failure

### Appendix C Quiz Answers

#### Quiz 1

*Instructions: A passing score for this section is 5 out of the 7 questions correct. The faculty facilitator will review all answers with your team. If the target score is achieved, you will be given a section of a puzzle to complete and the next quiz. If the target score is not reached, your team will have to purchase an item on the department tab from the store. The next puzzle section and quiz will only be given when the entire team is seated with the purchased item, and you have corrected your answers (you may electronically look up answers if it’s your second attempt).*

1. 1. A 24-year-old woman at approximately 40-weeks’ gestation arrives at the emergency department in active labor with the fetal head visible at the introitus with a nuchal cord. Which of the following actions should be prioritized?
  - **Gently reduce the cord over fetal head and proceed with delivery process**
  - Administer medication to accelerate the delivery process
  - Secure the cord in two places with clamps and cut between the clamps
  - Elevate the presenting fetal part and proceed to emergency cesarean section
2. 2. A 23-year-old G3P2 woman with a history of polyhydramnios presents to the emergency department with a precipitous delivery. The fetal head delivers easily, but then the head retracts tightly against the perineum. Which of the following is the next most appropriate step?
  - **Flex the mother's thighs sharply against her abdomen to widen the pelvic outlet and apply suprapubic pressure**
  - Insert a finger into vagina and hook around the inferior clavicle, applying caudal pressure until it fractures
  - Rotate the fetus’ anterior shoulder towards the back to disengage it from behind the maternal pubic bone
  - Attempt to rotate the posterior shoulder into the anterior-posterior diameter of the pelvis

For the following questions, match the name of the appropriate maneuver to its picture and description.

3. The Wood’s Maneuver
4. The McRoberts Maneuver
5. The Menticoglou Maneuver
6. The Gaskin Maneuver
7. The Rubin Maneuver

Image 1:

The birthing person is positioned on hands and knees, leaning forward onto their forearms or hands, and their knees are spread apart. This posture allows gravity to aid the descent of the baby through the birth canal, can widen pelvic outlet, and reduce pressure on perineum.

**Answer: 6. Gaskin Maneuver**

Image 2:

The birthing person is positioned flat on their back, with legs sharply flexed at the hips and pushed back toward shoulders. This positioning helps to widen the pelvic outlet by rotating the pelvis, which can sometimes free the impacted shoulder and allow the baby to be delivered. Providers may combine this maneuver with other techniques, such as suprapubic pressure to further facilitate the baby's delivery.

**Answer: 4. McRoberts Maneuver**

Image 3:

The healthcare provider places one hand on the posterior or anterior shoulder (whichever is more accessible) and applies pressure **to adduct the shoulders together**. The baby is then rotated 180 degrees to help deliver the posterior shoulder anteriorly.

**Answer: 7. Rubin Maneuver**

Image 4:

The healthcare provider rotates the posterior shoulder **counterclockwise** until it is delivered anteriorly.

**Answer: 3. Woods Maneuver**

Image 5:

The birthing person is usually lying on back in a standard delivery position. The provider hooks both of their middle fingers under the axilla of the posterior arm. Another provider gently flexes the head of the fetus towards the anterior shoulder. Gentle traction is used to guide the posterior shoulder along the curvature of the sacrum until the posterior shoulder is delivered.

**Answer: 5. Menticoglou Maneuver**

#### Quiz 2

*Instructions: A passing score for this section is 6 out of the 7 questions correct. The faculty facilitator will review all answers with your team. If the target score is achieved, you will be given a section of a puzzle to complete and the next quiz. If the target score is not reached, your team will have to purchase an item on the department tab from the store. The next puzzle section and quiz will only be given when the entire team is seated with the purchased item, and you have corrected your answers (you may electronically look up answers if it’s your second attempt).*

The following questions pertain to a **breech** presentation if cesarean is not immediately available:

1. The fetal sacrum should be in which position for an ideal vaginal delivery in breech presentation?
  - **Anterior**
  - Posterior
  - Oblique
  - Right sacrum anterior
  - Left sacrum anterior
2. TRUE OR FALSE? Traction should be placed onto fetal buttocks to deliver umbilicus
  - TRUE
  - **FALSE**
3. TRUE OR FALSE? Fetal legs usually deliver spontaneously
  - **TRUE**
  - FALSE
4. Which of the following is the optimal practitioner hand positioning for **delivery of fetal legs** in breech presentation?
  - Grasp both legs at ankles and deliver
  - Grasp one leg at ankle, deliver, then repeat for other leg
  - **Grasp both legs at popliteal fossa and deliver**
  - Grasp one leg at popliteal fossa, deliver, then repeat for other leg
5. When **shoulders** are being delivered in breech presentation, which of the following is the optimal practitioner hand/finger placement on fetus?
  - Thumbs on abdomen, hands on back
  - Thumbs on back, hands on abdomen
  - **Thumbs on sacrum, hands on pelvis**
  - Thumbs on sacrum, hands on abdomen
  - Thumbs on shoulders, hands on chest
6. When the **fetal head** is being delivered in breech presentation, which of the following describes the correct technique?
  - Maintain cervical FLEXION by placing one hand on the fetal occiput and shoulders, apply flexing pressure on the occiput, and place the fingers of the other hand on the infant’s MANDIBLE to aid in cervical flexion.
  - **Maintain cervical FLEXION by placing one hand on the fetal occiput and shoulders, apply flexing pressure on the occiput, and place the fingers of the other hand on the infant’s MAXILLAE to aid in cervical flexion.**
  - Maintain cervical EXTENSION by placing one hand on the fetal occiput and shoulders, apply flexing pressure on the occiput, and place the fingers of the other hand on the infant’s MANDIBLE to aid in cervical flexion.
  - Maintain cervical EXTENSION by placing one hand on the fetal occiput and shoulders, apply flexing pressure on the occiput, and place the fingers of the other hand on the infant’s MAXILLA to aid in cervical flexion.
7. To complete delivery, which is the correct direction to rotate fetal legs?
  - **Rotate fetal legs up and towards mother**
  - Rotate fetal legs down and towards the ground

#### Quiz 3

*Instructions: A passing score for this section is 8 out of the 10 questions correct. The faculty facilitator will review all answers with your team. If the target score is achieved, you will be given a section of a puzzle to complete and the next quiz. If the target score is not reached, your team will have to purchase an item on the department tab from the store. The next puzzle section and quiz will only be given when the entire team is seated with the purchased item, and you have corrected your answers (you may electronically look up answers if it’s your second attempt).*

1. Which of the following patients with renal colic meets the criteria for hospital admission based on their clinical presentation?
  - A 40-year-old woman with a 3 mm stone at the ureteropelvic junction, complaining of moderate flank pain that is partially relieved by analgesics, with no signs of infection or dehydration.
  - A 35-year-old man with a 6 mm stone at the renal pelvis, having intermittent colicky pain that responds well to oral pain medication, no hydronephrosis on ultrasound, and presenting with normal vital signs and urinalysis results.
  - A 28-year-old pregnant woman with a 4 mm stone at the ureterovesicular junction, reporting mild to moderate pain, normal vital signs, and a urinalysis showing 1+ blood.
  - **A 30-year-old man with a 5 mm stone at the ureterovesicular junction, experiencing uncontrolled pain despite intravenous pain medication.**
2. Which of the following microscopic findings is most characteristic of acute tubular necrosis (ATN) in renal biopsy specimens?
  - Hyaline cell casts
  - **Presence of granular or muddy brown casts in the tubular lumens**
  - Proliferation of inflammatory cells and fibroblasts within the interstitium
  - Diffuse thickening of the glomerular basement membrane with immune complex deposits
3. A 55-year-old male patient presents to the emergency department with severe left-sided flank pain, hematuria, and dysuria for the past 24 hours. He has a history of recurrent urinary tract infections and kidney stones. On physical examination, he appears uncomfortable, and his blood pressure is 140/90 mmHg. Laboratory tests reveal leukocytosis and microscopic hematuria. A non-contrast CT scan of the abdomen and pelvis is performed, showing a large, branching stone that fills the renal pelvis and extends into the calyces. Which of the following compositions is most likely responsible for the formation of the calculus described in this patient's CT scan?
  - Calcium Oxalate
  - Uric Acid
  - **Magnesium, Ammonium, and Phosphate**
  - Cystine
4. A 50-year-old woman presents to her primary care physician for a routine check-up. She has a family history of kidney disease and hypertension. On examination, her blood pressure is 150/92 mmHg. Laboratory tests reveal elevated serum creatinine and proteinuria. Renal ultrasound shows enlarged kidneys with multiple cysts bilaterally. Which of the following blood pressure medications is the most appropriate initial choice to start in this patient?
  - **Angiotensin-Converting Enzyme Inhibitor**
  - Beta-Blocker
  - Calcium Channel Blocker
  - Thiazide diuretic
5. A 34-year-old man with polysubstance use disorder presents to the emergency department complaining of a painful, prolonged erection that has lasted for four hours. He denies any recent sexual activity or trauma. On examination, his penis is erect and tender. The rest of the physical examination is unremarkable. His vital signs are stable. What is the most appropriate initial management for this patient's condition?
  - **Intracavernosal injection of alpha-adrenergic agonist**
  - Immediate consultation with urology for surgical exploration
  - Oral phosphodiesterase-5 (PDE-5) inhibitor
  - Ice packs to the perineum and scrotum
6. A 58-year-old man with end-stage renal disease presents to the emergency department with abdominal pain, fever, and cloudy peritoneal dialysate fluid. On examination, he is febrile, and abdominal tenderness is noted. Peritoneal fluid analysis is performed via paracentesis. Which of the following findings on peritoneal fluid analysis is most consistent with the diagnosis of spontaneous bacterial peritonitis in this patient?
  - **Peritoneal fluid polymorphonuclear (PMN) cell count >250 cells/mm^3^**
  - Peritoneal fluid glucose of <50 mg/dL
  - Peritoneal fluid culture yielding normal skin flora
  - Peritoneal fluid demonstrating LDH levels ⅔ the upper limit of normal
7. A 45-year-old man with a history of bipolar disorder and chronic kidney disease stage 5, secondary to polycystic kidney disease, presents to the emergency department after a suicide attempt by ingesting multiple medications. He is agitated, tremulous, and hypertensive. The toxicology screen reveals the presence of several drugs in his system. If ingested by the patient, which of the following drugs could be removed by hemodialysis?
  - Digoxin
  - Lorazepam
  - **Carbamazepine**
  - Metoprolol
8. A 28-year-old pregnant woman at 36-weeks’ gestation is brought to the emergency department after a motor vehicle accident. She complains of abdominal pain and contractions. On examination, she appears distressed, and her vital signs are stable. Abdominal palpation reveals tenderness and guarding. During the speculum examination, you notice bright red blood coming from the cervical os. Which of the following physical exam findings is most indicative of uterine rupture following trauma in this patient?
  - Uterine tenderness to palpation
  - Bright red blood from the cervical os
  - **Loss of palpable uterine contour**
  - Decreased fetal movement and heart rate
9. A 26-year-old pregnant woman at 38-weeks’ gestation is brought to the labor and delivery unit due to leakage of fluid from her vagina. She denies any recent trauma or discomfort. On examination, there is a pool of clear fluid in the vaginal vault. The patient reports that she has been feeling continuous leakage for the past hour. Which of the following characteristics of the fluid is most indicative of presence of amniotic fluid in this patient?
  - Yellow-brown color
  - Fishy odor
  - Acidic pH
  - **Presence of ferning pattern under a microscope**
10. A 30-year-old pregnant woman at 34-weeks’ gestation presents to the emergency department following a motor vehicle accident. She was a restrained driver and was involved in a frontal collision. She is experiencing abdominal discomfort. On examination, she appears anxious, her vital signs are stable, and there are no visible external injuries. What is the most sensitive clinical finding that raises suspicion for placental abruption in this patient?
  - Visible vaginal bleeding
  - Fetal bradycardia on Doppler
  - **Uterine irritability**
  - Maternal tachycardia

#### Quiz 4

*Instructions: A passing score for this section is 8 out of the 10 questions correct. The faculty facilitator will review all answers with your team. If the target score is achieved, you will be given the final section of a puzzle to complete. If the target score is not reached, your team will have to purchase an item on the department tab from the store. The final puzzle section will only be given when the entire team is seated with the purchased item, and you have corrected your answers (you may electronically look up answers if it’s your second attempt).*

1. A 65-year-old man with newly diagnosed end-stage renal disease (ESRD) is brought to the emergency department after a hemodialysis session. He underwent an extended hemodialysis session due to significant fluid overload. Shortly after dialysis, he developed a severe headache, nausea, vomiting, and confusion. His blood pressure is 180/100 mmHg, and he appears restless and disoriented. Which of the following medications is most appropriate to initiate in this patient?
  - Lorazepam
  - **Mannitol**
  - Furosemide
  - Acetaminophen
2. A 4-year-old child is brought to the pediatrician due to parental concern about a swelling in the abdomen. On examination, the physician palpates a firm mass in the left upper quadrant of the abdomen that does not cross the midline. The child's parents report that the mass has been gradually increasing in size over the past few weeks. The child has no history of trauma or recent illnesses, and there are no other notable symptoms. What is the most common presenting sign of this condition?
  - Hematuria
  - Abdominal pain
  - **Painless abdominal mass**
  - Fever and weight loss
3. An 86-year-old uncircumcised man presents to the emergency department from a nursing home for complaints of redness, swelling, and discomfort around the head of his penis. He denies any history of sexually transmitted infections and reports that he has trouble keeping his genitourinary area clean. On examination, there is erythema, edema, and discharge accumulating beneath the foreskin. The rest of the genital examination is unremarkable. His vital signs are normal. What is the most appropriate initial treatment for this patient's condition?
  - Topical corticosteroids
  - Oral antibiotics
  - **Topical antifungal cream**
  - Warm saline baths and gentle retraction of the foreskin
4. A 28-year-old woman presents to the clinic with dysuria, urgency, and increased frequency of urination for the past two days. She denies any fever or back pain. Urinalysis reveals the presence of leukocytes and nitrites. The patient has no known allergies and is not pregnant. Assuming no specific local resistance patterns, what is the most appropriate initial treatment for this patient's condition?
  - Oral fluoroquinolone antibiotics
  - Oral cefpodoxime
  - **Oral nitrofurantoin**
  - Intravenous ceftriaxone
5. TRUE OR FALSE? Ionizing radiation should be withheld during trauma assessment in pregnant patients to protect the fetus
  - TRUE
  - **FALSE**
6. A 28-year-old pregnant woman at 35-weeks’ gestation is admitted to the emergency department with symptoms of dizziness, lightheadedness, and hypotension (blood pressure 90/60 mmHg). She is conscious, but her skin appears pale and clammy. Fetal heart rate is within the normal range. The obstetrician is called for consultation. What is the most appropriate initial patient positioning to address hypotension in this pregnant patient?
  - Supine position with a wedge under the right hip
  - **Left lateral tilt**
  - Sitting upright
  - Trendelenburg position
7. A transvaginal ultrasound is performed on a patient pregnant at 34 weeks who presented to the ED complaining of abdominal pain after a low-speed motor vehicle collision. Her uterus is tender to palpation. An ultrasound was obtained which demonstrates a focal hypoechoic area behind the placenta and signs of fetal distress. This presentation is most suggestive of which of the following pathologies?
  - Uterine rupture
  - Intrauterine fetal demise
  - **Placental abruption**
  - Placenta previa
8. A 45-year-old man presents to the urology clinic with sudden-onset pain and swelling in his right testicle. He reports dysuria and a frequent urge to urinate. On examination, there is tenderness and warmth over the right epididymis. He is sexually active with other males. The patient's medical history is notable for a recent urinary tract infection. Assuming no specific local resistance patterns, what is the most appropriate initial treatment for this middle-aged man with symptoms suggestive of epididymitis?
  - **Oral levofloxacin and IM ceftriaxone**
  - Oral levofloxacin
  - IM ceftriaxone and oral doxycycline
  - Immediate referral for surgical exploration and drainage
9. A 30-year-old man with sickle cell disease presents to the emergency department with a painful, persistent erection lasting for 4 hours. Physical examination reveals a rigid, non-tender penis with no signs of injury. Initial attempts at conservative management, including ice packs and hydration, have failed to resolve the priapism. What is the most appropriate next step in managing this patient's priapism?
  - Intravenous administration of phenylephrine
  - Oral administration of pseudoephedrine
  - **Aspiration of corpus cavernosum**
  - Emergent dorsal slit procedure
10. A 60-year-old female with a history of multiple myeloma presents to the emergency department with nausea, vomiting, and severe joint pains. Her vital signs are notable for a blood pressure 190/110 mmHg, and laboratory results show a pH 7.20, serum potassium 6.2 mEq/L, BUN of 64, and a creatinine of 3.4. Her calcium level is 14. Which of the following is an indication for emergent dialysis?
  - Metabolic acidosis with a pH <7.20
  - Hyperkalemia >6 mEq/L
  - Altered mental status
  - **Presence of renal failure**
