# Supplementary material for: The EMazing Race: A Novel Gamified Board and Clinical Practice Review for Emergency Medicine Residents
Source: J Educ Teach Emerg Med. 2025 Oct 31;10(4):SG1–SG49. doi: 10.21980/J8.52075 (PMC12594469; doi:10.21980/J8.52075)
Supplement: Supplementary file 1 [file 10-4-SG1-AppendixD.pptx]

## Slide 1
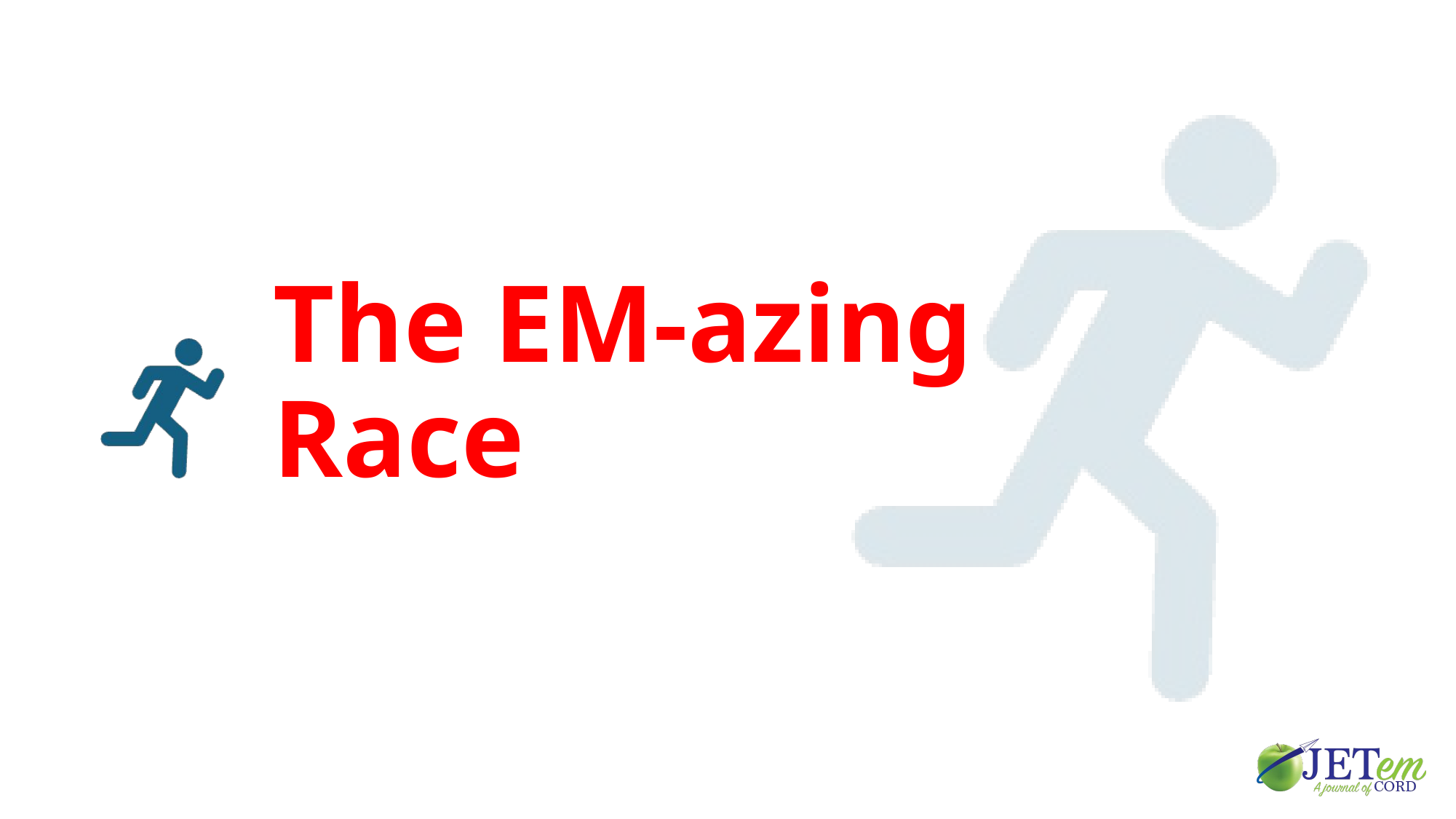

# The EM-azing Race

## Slide 2
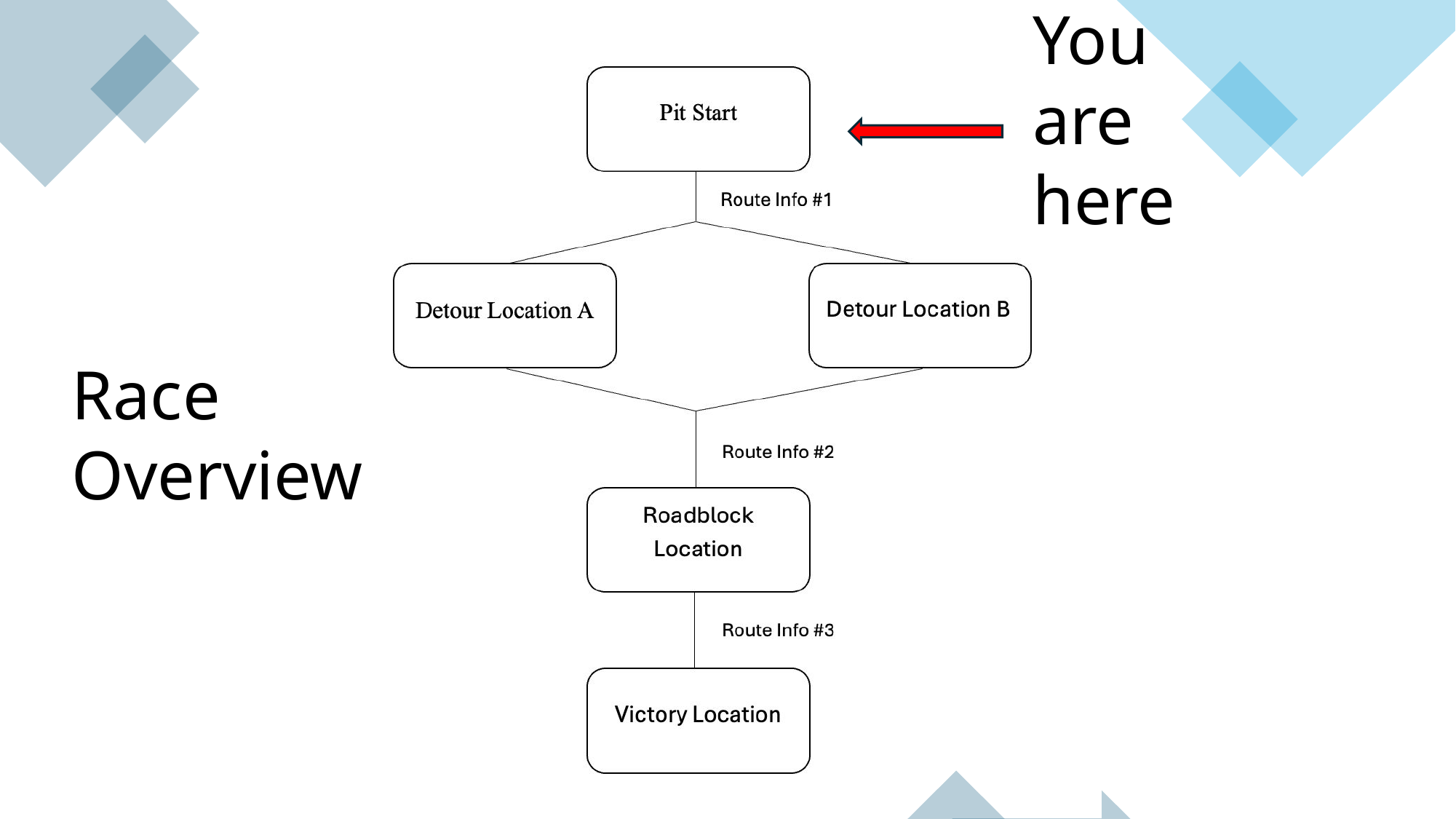

You are here
Race Overview

## Slide 3
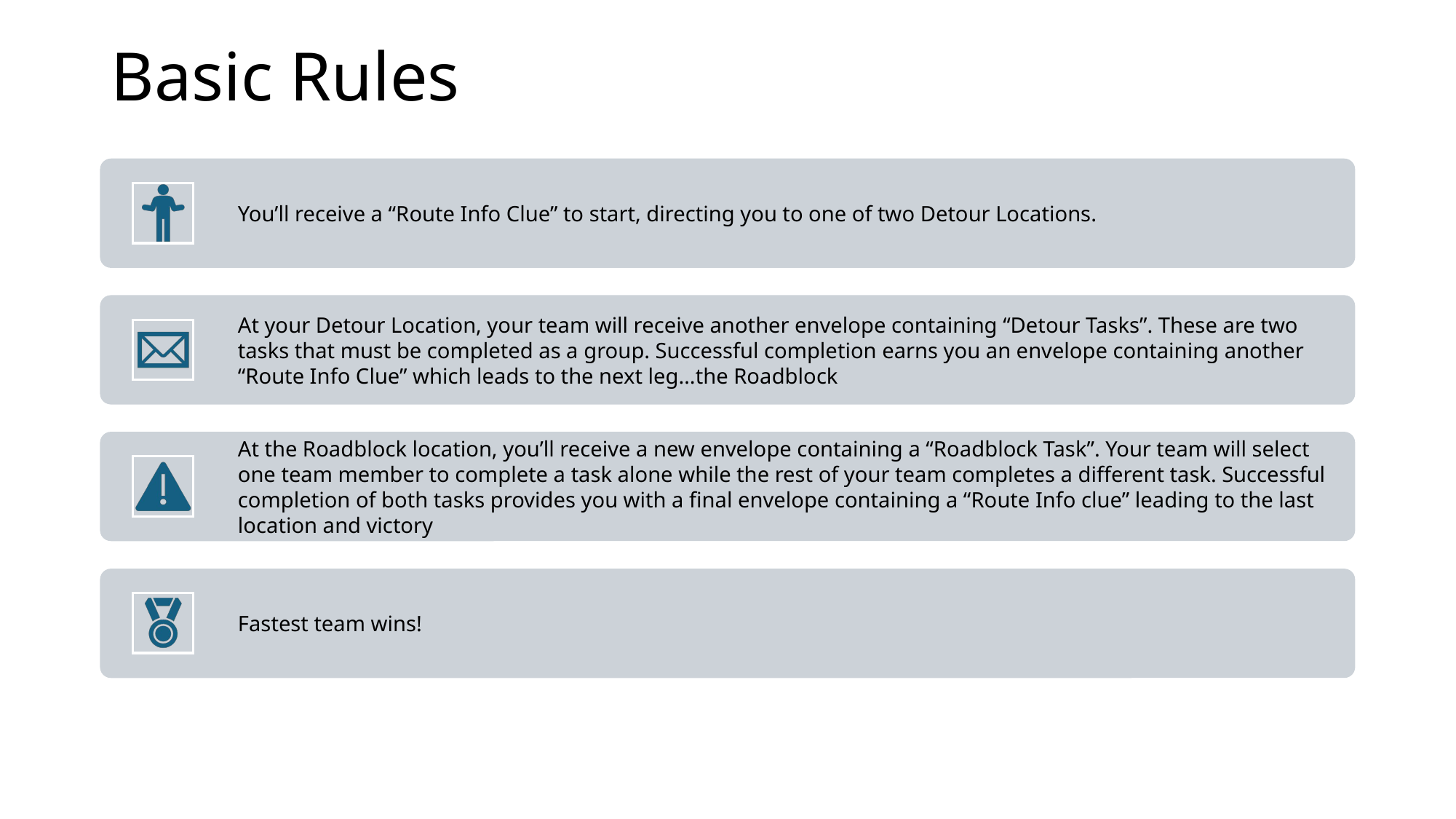

# Basic Rules

## Slide 4
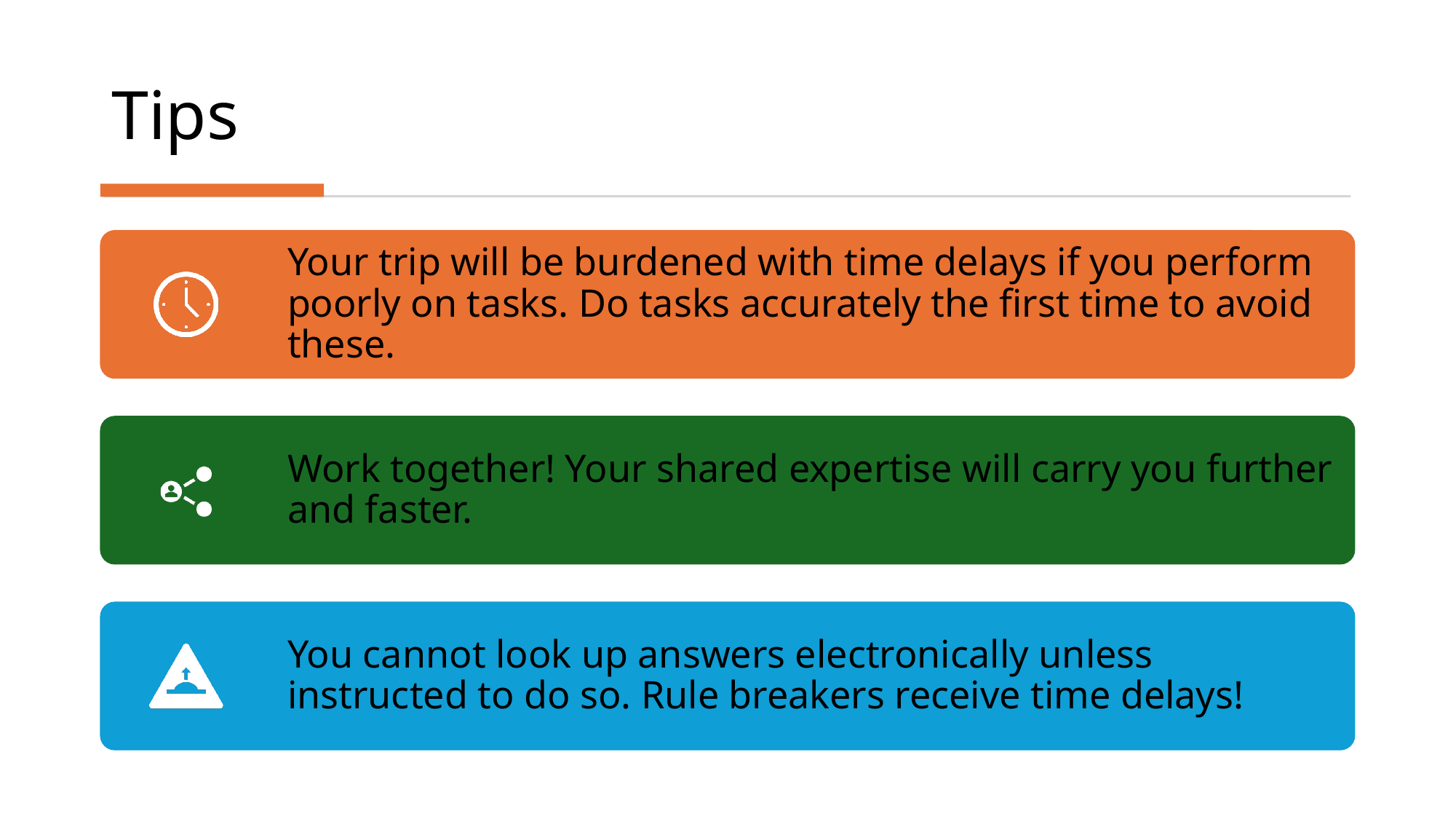

# Tips

## Slide 5
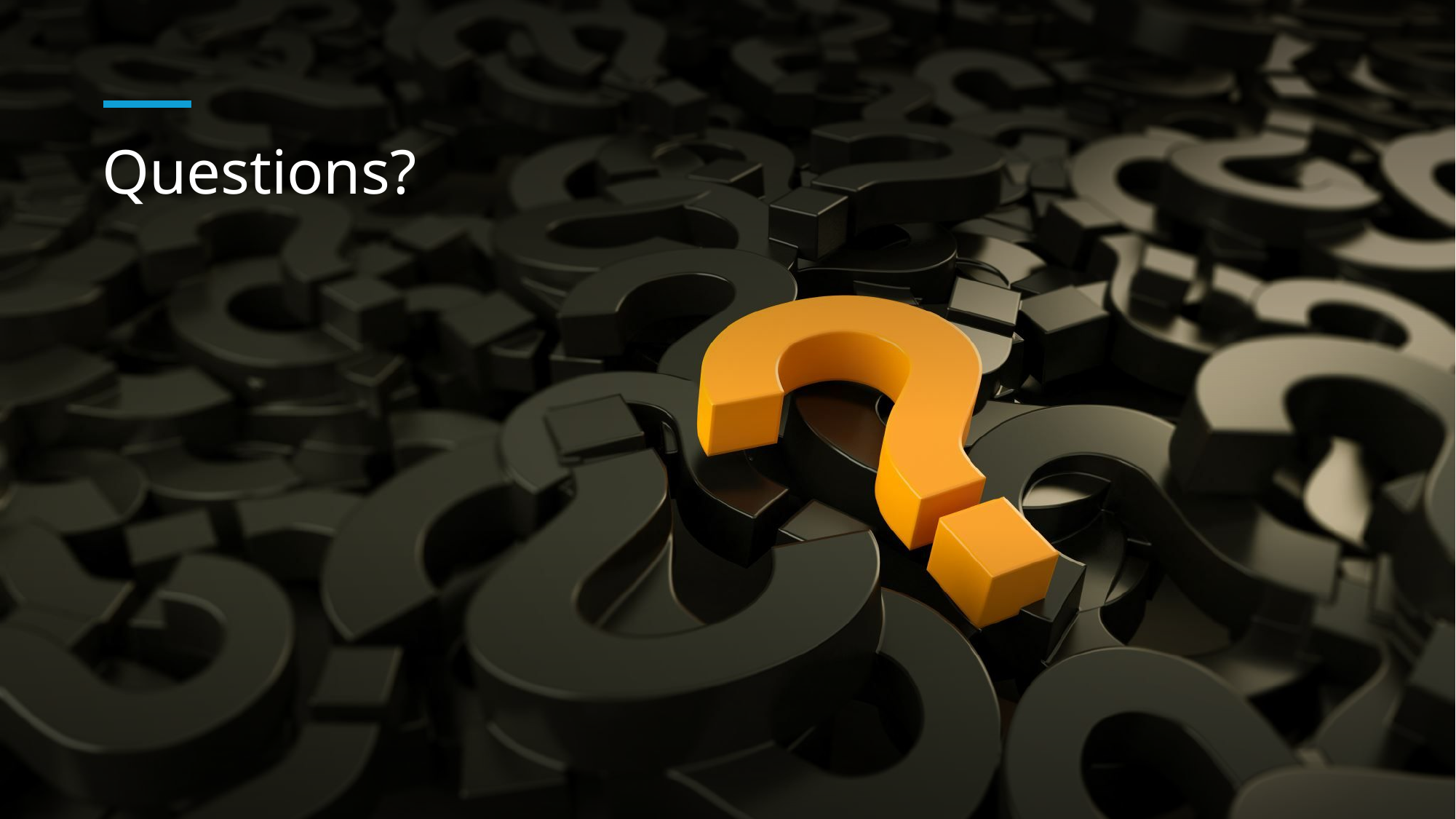

# Questions?
